# Orofacial Myofunctional Therapy for Feeding and Swallowing in Children From Four to 12 Years: What Does the Evidence Show? A Systematic Review

**DOI:** 10.1111/joor.70304

**Published:** 2026-09-08

**Authors:** Danielle Carey, Robyn Merkel‐Walsh, Phoebe Burrage, Melissa Hadlow, Ghidaa Hasan A. Alrehaily, Raymond J. Tseng, Sharon Smart

**Affiliations:** ^1^ North Carolina Tongue Tie Center Cary North Carolina USA; ^2^ Diamond MYO & Vocology Ridgefield New Jersey USA; ^3^ Brescia University Owensboro Kentucky USA; ^4^ TalkTools Charleston South Carolina USA; ^5^ School of Allied Health Curtin University Bentley Western Australia Australia; ^6^ Department of Pediatric Dentistry and Dental Public Health, Adams School of Dentistry University of North Carolina Chapel Hill North Carolina USA

**Keywords:** feeding, OMT, oral motor exercises, orofacial myofunctional therapy, swallowing

## Abstract

**Background:**

Orofacial Myofunctional Therapy (OMT), a subtype of oral motor therapy, applies neuromuscular re‐education techniques to address orofacial myofunctional disorders impacting breathing, sleeping, speaking, eating and swallowing due to atypical orofacial muscle function and structural anomalies. OMT is delivered by licensed professionals within their scope of practice. While some forms of oral motor therapy are passive and can be applied at any age, OMT is volitional and appropriate generally for children four and above.

**Objectives:**

The aim of this systematic review is to evaluate the effectiveness of OMT for improving feeding and swallowing outcomes in children aged 4–12 years and to evaluate the methodological quality of the available evidence.

**Method:**

This systematic review was conducted by searching four electronic databases for studies which analysed the use of OMT to treat paediatric feeding and swallowing disorders. Studies included children with a mean age of 4–12 years, reporting treatment outcomes after receiving OMT for feeding and swallowing difficulties. Seventeen papers were included for final analysis.

**Results:**

All 17 papers reported positive feeding and/or swallowing outcomes secondary to implementation of OMT; however, the quality of the selected studies was variable.

**Conclusions:**

The review highlighted positive clinical outcomes of OMT on feeding and swallowing and provided a compelling foundation for its continued clinical application. Future research employing rigorous, standardized study designs will be essential to definitively establish the treatment effectiveness and to formally position OMT as an evidence‐based intervention for paediatric feeding and swallowing disorders.

## Graphical abstract

This systematic review evaluated the effectiveness of orofacial myofunctional therapy (OMT) for improving feeding and swallowing outcomes in children aged 4–12 years. Searches of four databases identified 17 studies, all reporting positive feeding and/or swallowing outcomes, including improved eating and swallowing skills, orofacial muscle function and quality of life. Although findings suggest OMT is promising, high‐quality standardized research is needed to confirm its effectiveness as an evidence‐based intervention.

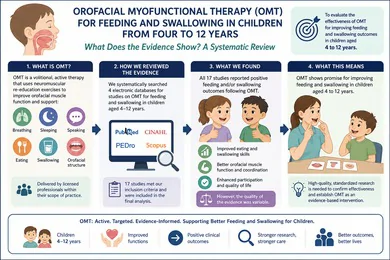

## 1 Introduction

Paediatric feeding and swallowing disorders or dysphagia, can compromise the safety and efficiency of swallowing, limit adequate oral intake and negatively affect growth, nutrition and overall health [1, 2]. Paediatric Feeding Disorder (PFD) describes a child with impaired oral intake that is not age‐appropriate and may be associated with medical, nutritional, suboptimal feeding skills and/or psychosocial dysfunction [3]. Studies show that up to 85% of children with neurodevelopmental disorders present with PFD, especially among children with developmental, neurological and structural conditions (e.g., cerebral palsy) [2, 3, 4]. A subset of children with PFD present with Orofacial Myofunctional Disorders (OMDs), which occur when atypical patterns of orofacial muscle function affect oral resting posture, breathing, eating, sleeping, speaking and swallowing and are accompanied by structural abnormalities [5, 6].

Oral phase dysphagia and OMDs have overlapping etiologies and symptoms, but the professionals who diagnose and treat them can be distinctly different [7]. Children may present with overlapping symptoms, such as challenges with mastication and bolus transport (Merkel‐Walsh [7]). OMDs are distinguished by structural anomalies of the oral and orofacial complex, often alongside developmental, neurological or comorbid pharyngeal or oesophageal disorders, whereas oral phase dysphagia is defined primarily by impaired bolus control and transit during the oral phase of swallowing itself. In addition, OMDs have a broader scope than dysphagia, encompassing airway, structure, sleep, breathing and speech, whereas dysphagia is a diagnosis limited to the oral, pharyngeal or oesophageal phases of swallowing. For this review, feeding encompasses the multifactorial process of food and/or liquid consumption, whereas swallowing refers specifically to the three physiological phases (oral, pharyngeal and oesophageal). The oral preparatory phase, including chewing, bolus formation and intraoral manipulation, represents an area where feeding and swallowing overlap.

Orofacial Myofunctional Therapy (OMT) is an individualized treatment program that aims to identify and extinguish maladaptive patterns secondary to an OMD. The primary purpose of orofacial myofunctional therapy (OMT) is to create an optimal oral environment to support typical orofacial functions, dental growth and reinforce structural stability [8]. OMT mainly targets the muscle‐based oral motor skills necessary for functional movement patterns, specifically oral resting posture, as well as nasal breathing, speech clarity and the oral phase of swallowing [7, 9]. OMT may also be implemented to improve sleep apnea, prevent orthodontic relapses, decrease noxious oral habits and alleviate the symptoms of temporomandibular joint dysfunction [10, 11].

OMT involves systematic, repetitive exercises (e.g., holding the tongue to the roof of the mouth for 20 s or completing structured lip‐ and cheek‐strengthening repetitions) and may be delivered in conjunction with orthodontic or myofunctional devices in response to a PFD [12]. OMT does not directly address all domains of PFDs including medical, nutritional and psychosocial domains [13]. For example, paediatric feeding therapy may target food acceptance with behavioural interventions [14], use adaptive feeding equipment, such as therapeutic cups or manipulate the placement of the food in the mouth [15]. As such, OMT and paediatric feeding therapy address different domains of PFDs, and the two can be viewed as complementary therapies, with OMT offered alongside PFT rather than as a substitute for it. Paediatric feeding also supports prevention of dehydration, aspiration and choking, which are not within OMT goals [16]. If a patient with an OMD requires swallowing support beyond the oral phase, a Speech‐Language Pathologist (SLP) with dysphagia expertise would be warranted [16].

OMT has emerged as an increasingly applied intervention to address PFD with oral phase swallowing disorders in children. OMT was initially developed in the early nineteenth century by orthodontists, Alfred Rodgers and Edward H. Angle and has since been adopted by professionals across disciplines [12, 17]. While OMT has grown as a popular modality, controversy persists. Disagreements stem from confusion in terminology and variations within a broader umbrella of ‘oral motor therapy,’ which includes oral motor exercises (OMEs), non‐speech oral motor exercises (NSOMEs), OMT and oral placement therapy [6, 9, 18], and disagreements on the quality and scientific validity of studies with different research designs in the current literature. These terms are frequently used interchangeably, despite differences in theoretical underpinnings, targeted ages and passive versus active implementation of exercises and neuromuscular techniques. Although there has been evidence that OMT can positively impact paediatric feeding and swallowing, study design and quality have led to scepticism. To date, no comprehensive systematic review has specifically examined the effectiveness and quality of evidence for OMT as a treatment modality for paediatric feeding and swallowing disorders. Given the growing clinical interest in OMT and the ongoing debate surrounding its efficacy, a focused synthesis of the available evidence is warranted.

The aim of this systematic review is to evaluate the effectiveness of OMT for improving feeding and swallowing outcomes in children with a mean age of 4–12 years and to assess the methodological quality of the existing evidence. For the purposes of this review, OMT is defined as an active, volitional therapy approach targeting the coordination, strength, endurance and functional integration of the orofacial musculature [7]. Unlike passive oral motor stimulation approaches, OMT requires deliberate execution of exercises, active self‐monitoring and clinician‐provided feedback. Since these skills typically emerge around 4 years of age, this review focuses exclusively on children with a mean age over 4–12 years.

## 2 Methods

A systematic review of the literature was conducted to extract, analyse and synthesize relevant literature. A meta‐analysis was not conducted due to the variability in study designs, measurable outcomes and participant characteristics, negating the possibility of a meaningful statistical analysis. The review was conducted in accordance with the Preferred Reporting Items for Systematic reviews and Meta‐Analyses (PRISMA) guidelines [19].

### 2.1 Search Methods

A title and abstract search were conducted for English language peer‐reviewed papers in the following four databases: CINAHL, ProQuest, Embase and Medline (Ovid) using the following search terms (Appendix A).

Papers retrieved during initial search were loaded to Covidence for title and abstract screening, based on the inclusion and exclusion criteria below. The use of Covidence as an online review tool permitted automation of duplicate studies, blind screening and conflict resolution [20]. Title and abstract screening were completed by two independent reviewers to ensure results were objective and consistent. Conflicts were discussed with SLPs with expertise in paediatric feeding, swallowing and OMT. Full text reviews were completed and conflicts resolved with input from the research team. Data extraction was completed through an excel spreadsheet via an iterative data extraction including quality appraisal and cross‐checked and agreed by group consensus.

#### 2.1.1 Inclusion Criteria

The included studies were peer reviewed, published in English and had children with a mean age of four to 12 with diagnosed feeding or swallowing disorder/difficulty. Studies were excluded if they included infants, children with a mean age younger than four or over 12 years or adults. Grey literature, study protocols and systematic or scoping reviews were excluded and studies not written in English or reporting outcomes related only to speech, sleep apnea, drooling, open bite were not included unless feeding and/or swallowing outcomes were also reported.

### 2.2 Quality Analysis

Articles were screened for quality with the Joanna Briggs Institute (JBI) tool [21]. The quality appraisal process was conducted by two independent reviewers, with conflicts resolved with a third reviewer through consensus discussion. Critical appraisal tools used included the checklists for randomized control trials, quasi‐experimental studies and case studies [22]. Each score was then converted to a percentage and categorized from ‘poor’, ‘adequate’, ‘good’ and ‘very good’.

### 2.3 Data Extraction and Synthesis

Data extraction was completed on Covidence and relevant information regarding population characteristics, study characteristics, outcomes and level of evidence recorded in a shared spreadsheet [20]. Discrepancies were resolved through consensus discussion with the project team. Given substantial variability across studies in outcome measure scoring protocols, follow‐up intervals and baseline participant severity, a meta‐analysis or pooling of effects was not used since it would likely produce a misleading composite result; instead, a qualitative synthesis approach was adopted.

## 3 Results

A total of 3850 studies were imported from the initial search. 2139 duplicates were removed by the Covidence software, with 55 removed manually. A total of 1658 studies were screened, with 1529 studies excluded, leaving 136 studies for the full text review. During full text screening, 119 studies were removed due to: interventions that were not strictly OMT [23], full text was not available [24], not peer reviewed. Reasons for exclusion included being a conference abstract [25], paper did not assess swallowing outcomes [26], investigated a population outside of the scope of this review [27] or was not written in English [28]. Seventeen studies remained for data extraction and quality appraisal. These results are summarized in Figure 1.

**FIGURE 1:**
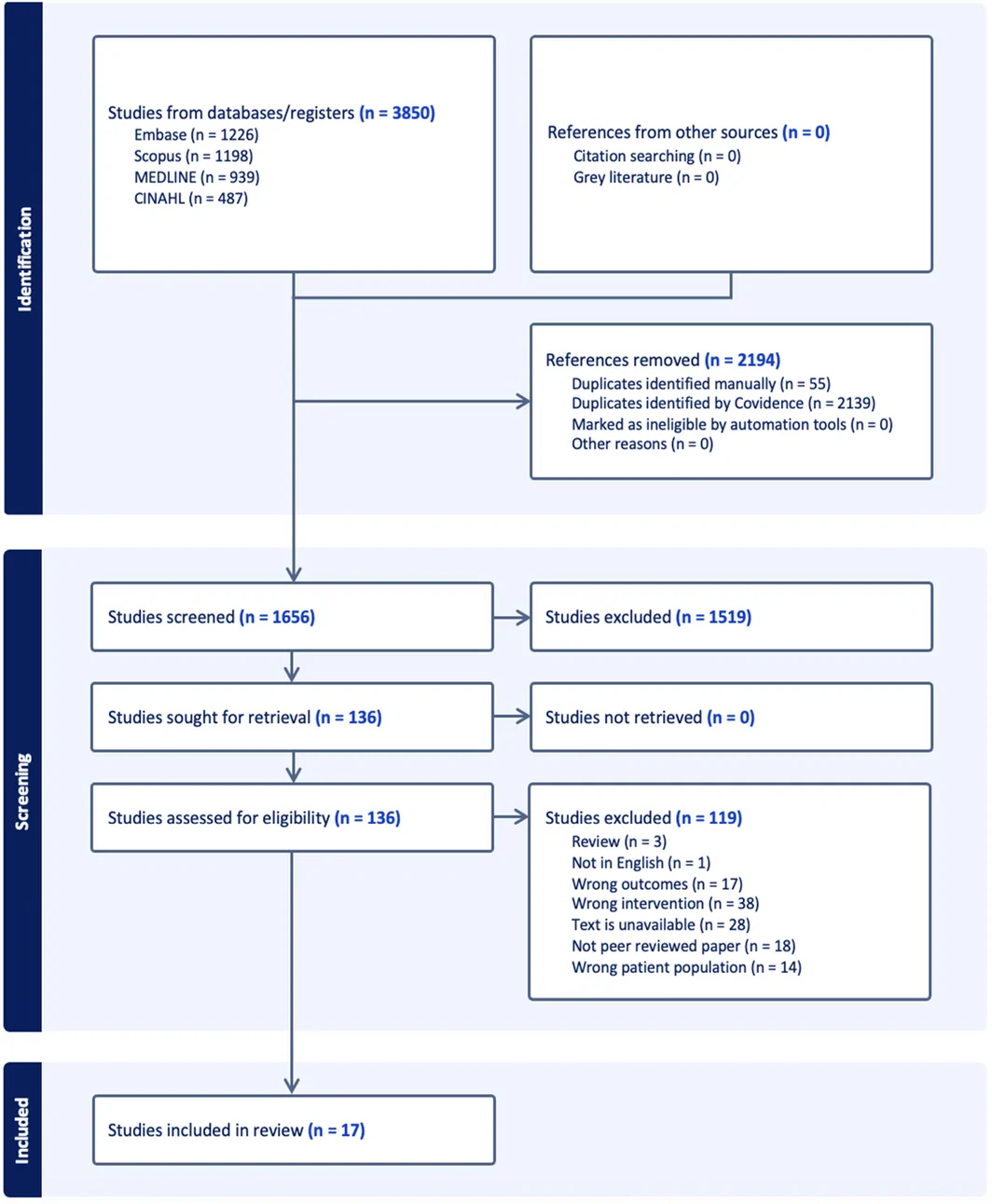
PRISMA Flowchart of Study Selection Process.

### 3.1 Study Design and Levels of Evidence

The review identified 17 studies that met the inclusion and exclusion criteria. Three randomized control trials (RCTs), 13 categorized as quasi‐experimental and one single case study [29]. According to Melnyk and Fineout‐Overholt's [30] classification of levels of evidence, this results in three studies being classified as Level 2, 13 as Level 3 and one as Level 6. Five studies had a control group that did not receive OMT; a further three studies had a control group that received OMT but differed from the therapy protocol received by the treatment group [30]. Table 1 outlines study design and level of evidence.

**TABLE 1:** Study classifications, participant characteristics, therapy modality and quality appraisal.

Author/Year/Country	Level of evidence	Study design	Participants	Participant background	Participants receiving OMT	Control group (C)/Alternate therapy group (ALT)	Untreated group	Combined with devices/other treatment	Did they define OMT?	Critical appraisal score
Achmad et al. (2022) [31], Indonesia	Level 3	Quasi‐experimental	27 children aged 7–13 years (mean age: 8.9 ± 1.3 = years)	Children with overjet > 4 mm	11 children aged 7–13 years (mean age: 8.9 ± 1.3 = years)	7 children aged 7–13 years (mean age: 8.9 ± 1.3 = years) with normal occlusion	9 children aged 7–13 years (mean age: 8.9 ± 1.3 = years) Who refused to use the pre‐orthodontic appliance (trainer)	Pre‐orthodontic appliance (Myo brace myofunctional trainer)	Referred to as ‘Myofunctional Therapy’. Myofunctional therapist stated that the proper function of the muscles on the oral cavity needs to be directed through exercises of the muscles of the oral cavity, to form a good occlusion (cited Rogers, 2011)	89%
Barber & Bonus (1975) [32], USA	Level 3	Quasi‐experimental	41 children aged 7.4–19.4. 24 males and 17 females	Group 1, 2 and 3 included children exhibited a tongue‐thrusting habit characterized by an anterior open bite, diastemata between the maxillary and mandibular anterior teeth and incompetent lips (a soft tissue morphology that does not permit the lips to seal with the mandible in rest position and in the absence of any active contraction of the orofacial musculature) or a combination of these conditions. Group 4 included children exhibited a normal swallowing pattern (no tongue‐thrusting habit), a normal overbite (no anterior open bite or diastemata between ‘e anterior teeth) and competent lips.	Treatment Group 1: 7 children aged 9.3–19.4 (mean age: 12.7). 5 males and 2 females for 6 months Treatment Group 2: 13 children aged between 7.4 and 12.1 (mean age: 9.1). 7 males and 6 females treated for 3 months	Group 3–12 children aged 7.4–11.6 (mean age: 9.8). 6 males and 6 females untreated Group 4–9 children aged 7.5–11.6 (mean 9.4). 6 males and 3 females untreated		No		56%
Baxter et al. (2020) [33], USA	Level 3	Quasi‐experimental	37 children (23 male, 14 females; mean: 4.2 years, age 13 months—12 years)	Children with speech, feeding and sleep issues	All participants			Lingual frenectomy with CO2 laser		44%
Cayley et al. (2000) [34], Australia	Level 3	Quasi‐experimental	8 males (range 9.08–11.75 years; mean 9.93)	Anterior open bite or incomplete overbite of **3** mm or more, tongue‐thrust	All participants			Trainer plate		42%
Dahan & Lelong (2003) [35], Belgium	Level 3	Quasi‐experimental	110 (age range, 7–12 years; mean, 9 years 8 months) 60 girls (mean age, 10 years 1 month) and 50 boys (mean age, 9 years 11 months).	Labiolingual dysfunction—(2 groups: weak + high)	All participants					67%
Giuca et al. (2008) [36], Italy	Level 3	Quasi‐experimental	57 children of which 31 were males (aged 5–13, mean age 8.2)	Children with atypical deglutition	All participants			Bionator		40%
Green (2013) [29]	Level 6	Case study	1 female (age 9 years)	Tongue thrust, posterior crossbite in conjunction with a significant anterior open bite, maxillary vertical growth propensity.	Yes					25%
Korbmacher et al. (2004) [37], Germany	Level 2	Randomized Controlled Trial	45 patients aged 3–16 years	Orofacial dysfunctions including: a division between ‘physiological’ and ‘pathologic’ swallowing pattern	MFT group (19 patients: 4 females, 15 male) Mean age: 8.4 years with myofunctional therapy FFT group (26 patients: 9 females, 17 male) The mean age: 8.3 years treated with myofunctional therapy and a face former appliance.			Face Former silicone appliance	Strengthening of the orofacial muscles to pave the way for mouth closure, establishment of nasal breathing and learning a physiological swallowing pattern.’	69%
Leal et al. (2025) [38], Brazil	Level 2	Randomized Controlled Trial	32 patients	Patients in mixed dentition phase with an open bite greater than 1 mm, complete eruption of maxillary and mandibular permanent central incisors and absence of posterior crossbite.	Group PC‐OMT: 16 children included 9 males, 7 females. Mean age 7.97 ± 1.00 years treated with fixed palatal crib and myofunctional therapy	Group PC: 16 children included 3 males, 13 females. Mean age 8.65 ± 0.93 years treated with fixed palatal crib only.		Fixed palatal cribs		80%
Overstake (1975) [39], USA	Level 2	Randomized Controlled Trial	67 children aged 7–12 years.	Children with (1) deviant swallowing, (2) open bite and/or overjet orthodontic problems and (3) interdental/s/speech sound difficulties.	Group 1 received swallow therapy only 0.38 male and female subjects aged 7–12 with deviant tongue‐thrusting swallowing.	Group 2 received swallow therapy and speech therapy. 38 male and female subjects aged 7–12 with deviant tongue‐thrusting swallowing.		Speech‐language pathology		46%
Ray (2001) [40], India	Level 3	Quasi‐experimental	16 children including 7 females aged between 7 and 10 years (mean age 8.6)	Mild to moderate cerebral palsy spasticity with either diplegia or hemiplegia, incoordination between respiratory, swallowing and speech movements. 12/16 participants had mild to moderate anterior open bite.	All participants				Program designed to focus on a lip‐closed, tongue‐in‐palate resting posture, along with facilitation of appropriate muscle movements of jaw, lips and tongue to facilitate accurate oral postures for articulation of speech sounds.	43%
Saccomanno et al. (2018) [41], Italy	Level 3	Quasi experimental	10 patients (7 males and 3 females), aged 9–18 years.	Down Syndrome with accompanying malocclusion, atypical swallowing, mouth breathing, muscular hypotonia, rhinorrhea, sialorrhea, snoring, dry lips and lip incompetence.	All participants			Labial button		30%
Saccomanno et al. (2019) [42], Italy	Level 3	Quasi experimental	6 children (5 males and 1 female), aged between 4.5 and 11.7 years (mean age of 7.7 years).	Altered lingual frenulum	Group 2 included 2 patients who were prescribed the home exercises according to the myofunctional therapy protocol to be performed daily without the supervision of the speech therapist.	Group 3–2 patients treated by the same speech therapist for 3 weeks, for 3 office visits per week and home exercises.	Group 1–2 patients with no treatment			44%
Saccomanno et al. (2024) [43], Italy	Level 3	Quasi experimental	Six patients, one female and five males, aged 7–14 years	A typical swallowing diagnosis of functional impairment (swallowing and oral breathing dysfunction)	Group 2—treated with traditional myofunctional therapy.	Group 1—treated with traditional myofunctional therapy exercises and edible tools.		Edible tools		77%
Scarano et al. (2023) [44], Italy	Level 3	Quasi experimental	130 children (76 female and 54 male) with ages spanning from 4 to 11 years (with an average age of 5.8 years)	Diagnosis of ankyloglossia based on Kotlow [45] classification Class III/IV and at least one of the following: observable and reported mouth breathing, observable dysfunctional swallow pattern, reported snoring, clenching and/or myofascial pain or tension.	All participants			Postural bench		44%
Schievano et al. (1999) [46], Brazil	Level 3	Quasi‐experimental	13 children aged 5–10 years	Open‐mouth posture. No known nasal obstruction, mouth‐breathing children with lower lip everted and tension in chin region during lip closure.	All participants					44%
Van Dyck et al. (2016) [47], Belgium	Level 3	Quasi‐experimental	22 children (11 males, 11 females; age range: 7.1–10.6 years)	AOB and visceral swallowing pattern.	10 children who were divided into two groups: COMBI subgroup first underwent 10 h of OMT, followed by a treatment with a removable expansion device (*n* = 4, mean age 8.4, age range 8.1; 8.7) MYO subgroup underwent 10 h of OMT, during 10–20 sessions of 30–60 min (*n* = 6, mean age 8.3, age range 7.1; 9.3)	Device subgroup. Mean age 8.7, (age range 7.5: 9.8) treated with a removable expansion device only	6 children, mean age 9.1, (age range 7.7: 10.6)	COMBI group: Used acrylic resin plate		89%

Publication dates ranged from 1975 to 2025. Two papers were published in the 1970s, one in the 1990s, five in the 2000s, four in the 2010s and five in the 2020s (Table 1). Studies were conducted in multiple countries, including Italy (31.25%), United States (18.75%), Brazil (12.5%), Belgium (6.25%), Indonesia (6.25%), Australia (6.25%), India (6.25%) and Germany (6.25%). One paper had no identifiable origin of publication (Green [29]).

### 3.2 Participants

Across the seventeen studies, a total of 633 participants were reported, with 255 male, 234 female and 144 of unknown gender. Of the 633 participants, 544 children received some form of OMT (219 male; 211 female; 114 unknown gender). The mean age of the children ranged from four to 18 years. The average study population was 42.2 children, with the largest sample size being 130 [44].

Study populations presented with a wide variety of swallowing dysfunctions, including tongue thrust (*n* = 4), atypical swallowing (*n* = 3), swallowing challenges secondary to open bite or malocclusion (*n* = 4) and general feeding difficulties as determined by the researchers. Studies were also conducted on specific populations, such as cerebral palsy [40] and Down syndrome [41]. Full participant characteristics are outlined in Table 1.

### 3.3 Quality Appraisal

The quality of the studies was rated as follows: ten studies were appraised to be ‘poor quality’ (< 50%); one study was appraised as ‘fair’ (50%–60%); three studies were appraised as ‘good’ (60%–75%); and three studies were appraised as ‘high quality’ (> 75%) (Table 1) [22].

### 3.4 Treatment and Outcomes

A wide variety of healthcare professionals were reported to participate in the delivery of orofacial myofunctional therapy (OMT). These professionals included speech‐language pathologists (SLPs), registered dental hygienists (RDHs) orthodontists, surgeons, occupational therapists (OTs) and physiotherapists (PTs). SLPs were the most frequent health professional reported to use OMT as a therapeutic modality, being referenced in 67.7% of papers (11/17) (Table 2).

**TABLE 2:** Summary of swallowing processed treated, professionals involved in treatment and overview of treatment.

Author/year/country	Swallowing processes/functions targeted	Professionals involved	Overview of treatment
Achmad et al. (2022) [31], Indonesia	Swallowing, chewing, breathing and speech functions	Trained dentists	A removable intraoral device was used for 2 h daily and during sleep. Myofunctional exercises were carried out two to three times a day. The pre‐orthodontic appliance was used for 2 h daily and overnight.
Barber & Bonus (1975) [32], USA	Lip seal	Not reported	Lip strength is targeted by pulling on a spring scale with lips. The exercise was performed twice daily (morning and afternoon) for 15 min per session, totaling 30 min of exercise per day
Baxter et al. (2020) [33], USA	Tongue elevation in feeding	Speech Pathologist, Dental Hygienist	Not provided
Cayley et al. (2000) [34], Australia	Tongue thrust	Speech Pathologist	Therapy consisted of two stages. The first was directed towards developing new muscle patterns to support correct lip and tongue postures. The second was directed towards instruction and practice in normal swallowing using liquid and semi‐solid materials. Four 20‐min sessions were conducted, spaced fortnightly.
Dahan & Lelong (2003) [35], Belgium	Tongue thrust during swallowing	Students, technicians and teachers from the High School of Speech Therapy and the University of Louvain	Children performed various tasks with a thin layer of chocolate paste painted evenly on the tip of the tongue or on the occlusal surfaces of the mandibular arch, right side.
Giuca et al. (2008) [36], Italy	Tongue function and position, swallowing	Not reported	Children performed daily exercises progressing from sensory awareness (tongue brushing and pressure work) to motor control (dorsal and inferior pressure), postural correction (cooling and rubber bands to position the tongue) and automatization (making normal swallowing spontaneous). Some children with severe malocclusion also used a bionator. 8 sessions were conducted.
Green (2013) [29]	Tongue thrust	Orthodontist Orofacial Myofunctional Therapist	Tongue, lip and jaw rest posture techniques were used. The child and family were educated on the appropriate ‘spot’ location for the tongue, the concept of freeway space in relation to rest position, limited vertical tip and mid tongue elevation strategies, behavioural and environmental cues for conscious thought of rest posture and retraining exercises to encourage vertical tongue propensity.
Korbmacher et al. (2004) [37], Germany	Overall swallowing	Speech Pathologists and Orthodontists performed assessments; a Dental Assistant conducted FFT under Dentist supervision.	Face Former therapy (FFT) uses a silicone appliance to perform tongue and lip exercises, including lip compression and relaxation, to improve facial muscle tone. The appliance is worn for 6 weeks, with follow‐up conducted every 6 weeks. The control group received ‘conventional’ OMT (not defined).
Leal et al. (2025) [38], Brazil	Tongue pressure during swallowing	Speech Pathologist	Therapy focused on orofacial rehabilitation and muscle strengthening through structured exercises. Therapy was delivered via an illustrated booklet and included nasal hygiene, lip and tongue mobility exercises, tongue strength training, functional nasal breathing training, mastication and swallowing training, followed by speech articulation exercises, with online games to support practice.
Overstake (1975) [39], USA	Anterior tongue‐thrust, overall swallowing	Undefined ‘specialist’	Normal swallowing was taught by positioning the tongue and mandible in their ‘proper postures’ before initiating reflex swallowing. Swallow practice was to be done for 5 min before each meal using half a glass of water, milk or juice; parents were given a home program of instructions for swallowing therapy. Practice on different kinds of food was added during therapy, progressing from semi‐solids to difficult‐to‐swallow food. Each child was on a variable time schedule for therapy. Sessions were 15 min and scheduled at 1‐, 2‐, 3‐, 4‐, 5‐, 6‐, 8‐ and 10‐week intervals, extending over a nine‐month period.
Ray (2001) [40], India	Lip seal, rest posture and mobility of tongue lips and jaw.	Speech Pathologist; assisted by Occupational Therapist and Physical Therapist. Orthodontist verified resting postures	Therapy was carried out in six phases: awareness and education, tongue tip exercises, posterior tongue exercises, labial seal, tongue resting posture and jaw exercises. Therapy was provided 5 days a week for 25 min (15 min individual, 10 min group) over 4 months. Parents were instructed to carry out exercises 4–5 times daily.
Saccomanno et al. (2018) [37], Italy	Tongue rest position, lip seal, function of buccinator and masseter muscles, strengthening the soft palate, overall swallowing	Speech Pathologist	Therapy was administered for 20 weeks. Each child is given 2–3 target goals with accompanying exercises. Children repeated exercises at home 3× per day or for 30 min.
Saccomanno et al. (2019) [42], Italy	Tongue rest posture and elevation, oral proprioception, mouth opening, coordination of the orbicularis oris, lip seal, oral musculature strength and endurance, pharyngeal phase of swallowing	Speech Pathologist	Children were assigned 8 exercises (2 per goal), which were repeated 15 times in three sessions per day in front of a mirror. Each day the children performed at least 30 min of myofunctional home therapy. All children completed a therapy diary. 2 children received 3 weeks of therapy, with a frequency of 3 office visits per week, plus home practice.
Saccomanno et al. (2024) [43], Italy	Overall swallowing, bolus preparation, masticatory and lingual muscle reinforcement and proprioception of oral cavity	Speech Pathologist	The treatment group replaced traditional appliances with food, such as solid candy, spreadable cream, Nutella and cookies. A full protocol of the OMT program was provided in the appendices. Therapy was 6 months of 12 30 min OMT sessions, with 15mins of home practice. The control group used OMT with tactile tools to target disordered swallow patterns and the strength and co‐ordination of the lingual and peri‐oral muscles.
Scarano et al. (2023) [44], Italy	Tongue mobility, chewing and overall swallowing. Co‐ordination of jaw muscles during speech	Speech Pathologist, Surgeon	All patients received sessions of OMT before and after lingual frenectomy. Therapy was conducted over 6 months for 10 sessions, which varied according to individual cases. The average duration of time from treatment date to follow‐up was 6.3 ± 3 months, ranging from 4 to 9 months.
Schievano et al. (1999) [46], Brazil	Respiration, chewing, swallowing, speech/voice, plus rest posture of lips/tongue/cheeks/chin	Dentist, Speech Pathologist	Isometric exercises were conducted for lips, tongue, chin region and chewing muscles, facial massage (esp. chin region) and feeding re‐education. Parental supervision was utilized for home practice. Therapy was 9 months with weekly 30 min sessions.
Van Dyck et al. (2016) [47], Belgium	Tongue posture	Speech Pathologist	Stages of therapy included; Strengthening tongue and lip musculature, basis of swallowing process targeted, strengthening the anterior mid and posterior part of the tongue, co‐ordination of the total swallow movement, practicing conscious and unconscious habit formation, control of physiological swallowing act and follow‐up.

The type and administration of OMT varied. Individualized OMT therapy targets orofacial musculature and involves repetitive, muscle‐based exercises aimed at developing oral motor skills that produce functional movement patterns. Eight studies used OMT combined with a myofunctional device or therapy tools [31, 35, 36, 37, 38, 40, 43, 47], while another three combined OMT with other therapy or surgery, including speech therapy [39] or lingual frenectomy surgery [33, 44], with all 11 studies reporting improvement on their respective outcome measures (see Table 1 for individual study details). Six studies that conducted OMT in isolation [29, 32, 34, 41, 42, 46] reported improvements on their targeted measures. The specific nature and magnitude of improvement (partial versus full resolution) varied across study and outcome measures. Schievano et al.'s paper reported muscle and functions improved after OMT, with improved lip closure, muscle strength and improved mouth breathing in clients without airway obstruction [48]. Saccomanno et al. [41] investigated OMT in children with Down Syndrome and found that when OMT therapy protocols were individualized and performed correctly, OMT resulted in increased strength of the orbicularis oris and improved lip closure, improved awareness of correct posture for breathing and swallowing and reduced drooling and nasal secretions. Green [29] also reported similar oral phase improvements with improved tongue elevation and swallowing patterns post elimination of a thumb sucking habit with myofunctional therapy. Taken together, these studies show that OMT is effective in restoring orofacial functional outcomes when therapy is individualized in a variety of patient populations.

Saccomanno et al. [42] separated participants into three groups: no treatment, home exercise OMT protocol without the supervision of an SLP and OMT protocol with the supervision of an SLP with sessions three times a week and home practice. The third group acquired appropriate oral resting position, had better awareness of their oral cavity and demonstrated an increase in Tongue Range of Motion Ratio (TRMR) measurements, 50% of participants in Group 3 demonstrated increased tongue strength measured by the Iowa Oral Performance Instrument (IOPI), 100% of participants in Group 3 had improved swallowing function on clinical assessment and demonstrated improved breathing function [42]. Similarly, in 2024, Saccomanno and colleagues compared two active OMT protocols and separated participants into two groups. One group received traditional myofunctional therapy using conventional tools while the second received the same OMT protocol using food (e.g., spreadable cream and small candies) as the therapy tool [43]. Both groups received 12 weeks of OMT and demonstrated improved swallowing function, indicating that the delivery method of OMT, rather than OMT itself, was variable under comparison. The same SLP treated both groups and reported that the food‐based group had a higher level of motivation and cooperation and greater improvement in upper and lower lip protrusion [43].

Several studies used other terms besides OMT to describe the therapy conducted, and only three studies provided a definition of OMT or referred to a specific definition or previous program from the literature (Table 1) [34, 35, 37]. Many studies omitted details on therapy dosage and frequency, therapy protocol or a basic description of the exercises performed. Due to these factors, objective comparison across studies was challenging due to variability in reporting. Full details of therapeutic procedures, dosage and frequency are outlined in Table 2.

A variety of structures and functions related to swallowing were investigated. While many of the seventeen included studies investigated disordered swallowing as a primary outcome, one study reported swallowing function as a secondary measure [40]. A wide variety of methods to measure swallowing function were used. Seven studies measured swallowing outcomes using only subjective measures (e.g., parent report or rating scale) [29, 33, 35, 36, 39, 40, 44], four used objective measures [31, 32, 34, 38] and six used a combination of objective and subjective measures [37, 41, 42, 43, 47, 48]. A summary of swallowing outcomes is outlined in Table 3.

**TABLE 3:** Summary of feeding and swallowing outcomes and measurement instruments.

Swallowing/feeding outcomes	Objective measures	Subjective measures
Improved swallowing patterns	IOPI [38] Fluorescein [41]	Clinical examination [37, 39] Clinical examination with photographic documentation [41, 42] Assessor examination [47] Caregiver report [40] Clinical Observation and caregiver report [29] Unclear/assessor report [36]
Improved tongue strength, posture, sensitivity and/or mobility	Cephalometric radiographs, dental study casts [32] Electrophotography [34] IOPI [38, 42, 47] Fluorescein, dynamometer, photogrammetry [43]	Clinical examination [35, 39] Clinical examination with photographic documentation [29, 42, 43] Unclear/assessor report [36]
Improved lip control	Spring scale [32] Myo‐Bar‐Meter [37] Fluorescein, dynamometer, photogrammetry [43] IOPI [42] Dynamometer [41] Surface EMG (RMS) [48]	Clinical Examination [39] Clinical examination with photographic documentation [41, 43]
Facial morphology and functions		Clinical Examination [48] Clinical examination with photographic documentation [29]
Improved bite control	Cephalometric radiographs [34, 38]	Clinical Examination [35]
Soft palate mobility/tongue–palate contact	Cephalometric radiographs [34] Palatography [37]	Clinical examination with photographic documentation [41]
Improved chewing time or function		Clinical examination [40] Clinical examination with photographic documentation [41]
Improved feeding		Parent Questionnaire [33, 44]
Contraction strength in the temporalis, masseter and mentalis	Electromyography [31]	Clinical examination [39]
Bolus manipulation		Clinical examination [40]

## 4 Discussion

The overall findings indicate that OMT is an effective therapeutic modality for children aged four to 12 years of age, with all included studies reporting improvements in feeding and swallowing outcomes. Benefits spanned both structural domains, such as enhanced tongue strength and mobility and functional domains, including improved feeding performance. These results suggested potential benefits of OMT including improvements to structural (e.g., tongue strength/mobility) and functional (e.g., feeding) outcomes. The positive outcomes highlight the potential of OMT to remediate the oral phase dysphagia related to orofacial myofunctional disorders in paediatric populations. Effective swallowing requires precise motor planning and coordinated execution across oropharyngeal structures, including the tongue, lips, jaw, cheeks and soft palate [12, 46]. Targeted orofacial exercises targeting these oral movement patterns appear to improve functional gains in children with atypical swallowing.

Although positive outcomes were reported across studies, the quality of the evidence base constrains the extent to which firm conclusions can be drawn regarding the effectiveness of OMT. Of the study designs included in this review, many did not include a control group and included small sample sizes. A similar published review also noted that nearly half of the OMT literature has used single‐arm study designs [9]. This limits the ability to extrapolate the positive conclusions of the studies and present a clear statement on the effectiveness of OMT in treating feeding and swallowing dysfunction. Without a control group, it is difficult to confidently attribute improvements to the intervention as spontaneous recovery cannot be ruled out. This is reflected in critical appraisal, with 65% of the studies being rated as ‘poor’. OMT was frequently combined with other treatments/therapies such as orthodontic appliances or surgery for tongue tie (frenectomy), limiting the ability to attribute positive conclusions and improvements to OMT alone. This does not diminish the individual functional improvements achieved by these participants; rather, it highlights the need for larger, well‐designed studies to more thoroughly evaluate treatment outcomes regarding OMT and paediatric feeding disorders.

A critical limitation of this literature is the scarcity of naïve control groups against which OMT's isolated effects can be evaluated. Only three included studies were randomized controlled trials [37, 38, 39], none of which employed a true no‐treatment arm; instead, comparators consisted of appliance‐based or multimodal conditions. Barber and Bonus [32] and Van Dyck et al. [47] included untreated or device‐only subgroups, respectively, though without randomization. Where controlled comparisons existed, OMT demonstrated meaningful additive benefit: Overstake [39] found that children receiving swallowing therapy alone achieved significant improvements in deviant swallowing patterns and interdental sibilant production, with the addition of speech therapy yielding further gains in articulatory accuracy, collectively demonstrating that targeted myofunctional intervention produced outcomes exceeding those attributable to maturation in untreated peers. Leal et al. [38] found that children receiving fixed palatal crib combined with OMT achieved significantly greater tongue pressure normalization and open bite correction compared to those receiving the crib alone, with OMT emerging as an independent predictor of successful bite correction. Van Dyck et al. [47] similarly reported that children in the combined OMT‐plus‐device group demonstrated superior improvements in tongue posture and swallowing pattern compared to the device‐only subgroup, with the OMT‐only subgroup also outperforming device‐only peers on visceral swallowing resolution. Together these findings suggest a genuine treatment effect beyond appliance‐mediated or developmental change alone; however, the predominance of quasi‐experimental single‐group designs across this review means maturation, regression to the mean, and natural developmental progression cannot be excluded as competing explanations. Waitlist‐ or sham‐controlled trials are needed to establish causal efficacy. It is worth noting that OMT, like many individualized rehabilitation therapies, is not easily reduced to a fixed, standardized protocol used in classic randomized control trial designs, as treatment is deliberately adjusted in response to each child's presentation and progress. This does not remove the need for stronger controlled designs, but it may explain the predominance of quasi‐experimental methodology in this literature to date.

Regarding whether adjunctive interventions were associated with better outcomes than OMT alone: Three studies with controlled comparisons suggest OMT contributed additive benefit beyond device‐only or untreated conditions, though the review cannot formally test this trend given the absence of a no‐treatment comparator in most studies. In over half of the studies included (64.7%, 11/17), OMT was not delivered in isolation but as part of a multidisciplinary treatment plan. This highlights the complexity of treating OMDs and the need for multifactorial interventions. Eight studies incorporated OMT with a device such as intraoral orthodontic devices [31] or post‐frenectomy rehabilitation [33]. In these cases, it is difficult to separate from the impact OMT had on the outcomes, as the improvements could result from the devices or surgical interventions due to the lack of control group. Without the use of randomized control groups or cohort studies to isolate OMT as a standalone variable, it remains unclear whether improvements stem from OMT, the treatment/therapy it was combined with or both together. This constraint was highlighted in prior reviews, which emphasized the need to employ the use of control groups to better understand the contribution of OMT to clinical outcomes [9, 12].

Significant variability in outcome measurement and analytical rigour across the included studies further limits the interpretability of findings. Although 10 of the 17 studies (59%) performed some form of statistical analysis, none reported effect sizes, making it impossible to assess the clinical significance or magnitude of the observed changes and precluding any determination of whether reported improvements represented partial or full resolution of presenting symptoms. Furthermore, most outcome measures used across these studies were not standardized, psychometrically validated instruments with established sensitivity to clinically meaningful change; many instead relied on clinician‐rated or observational measures of swallowing pattern, tongue posture or lip seal. In studies that included statistical analyses, many feeding and swallowing outcomes relied predominantly on subjective measures. When objective measures were included, improvements were noted, but no statistical analysis or long‐term follow‐up was included to validate the outcomes and the applicability of the findings. While heterogeneity in outcome measures is not uncommon in clinical research, the degree of variation across studies in this review complicates cross‐study comparison, which is not atypical in the clinical setting. Each study used different measurement tools (both subjective and objective) and measured different outcomes of multiple orofacial structures. This heterogeneity across measurement tools, target structures and outcomes prohibited meaningful synthesis and identification of consistent treatment effects.

SLPs were the most frequently reported providers of OMT across the included studies (67.7%, 11/17), and one study found that feeding outcomes were more favourable when therapy was delivered under SLP supervision compared with unsupervised home practice [42]. This finding suggests that clinical oversight may influence treatment response, though the small number of studies directly comparing provider type limits the strength of this conclusion. Future research comparing outcomes by the type of provider is required to examine this relationship further. Many studies did not provide thorough reports/guidelines regarding how therapy was delivered, and other crucial information such as dosage, frequency, duration and what exercises were performed, thereby impeding reproducibility of the study and generalizability of the findings. In some studies, it was not clear whether the professionals delivering the therapies were licensed and/or certified professionals, and there could be a lack of expertise with paediatric dysphagia to properly provide therapy and measure treatment outcomes.

This review aligns with the findings from previous reviews that the evidence base for OMT remains vague and that generalizability of the findings remains difficult [9, 12]. While the effectiveness of OMT is reported, the limited quality of the evidence makes it difficult to make reliable conclusions. We note the related work of Sjögreen and colleagues [49] who examined a broader set of outcomes including speech, drooling and bite alongside feeding; these were not eligible for inclusion in the present review as narrative rather than systematic reviews. Despite these limitations, this review acknowledges the growing body of evidence of the potential effectiveness of OMT in managing feeding and swallowing dysfunction in children. SLPs possess specialized training in the anatomy, physiology and neurology of the orofacial complex, equipping them with the clinical expertise necessary to assess and treat feeding and swallowing dysfunction across the lifespan. Saccomanno et al. [43] highlighted that feeding outcomes were better when therapy was delivered by an SLP, emphasizing the importance of specialized professional involvement in facilitating orofacial exercises.

Future research should prioritize the use of well‐designed studies, incorporating appropriate control or comparison groups to strengthen the evidence base. Standardizing the definition of OMT and ensuring detailed protocol reporting, including exercise type, dosage and treatment duration, are critical steps towards improving conceptual clarity and replicability. Future studies would benefit from examining the effects of age on treatment initiation on short and long‐term outcomes to identify the optimal age to initiate OMT. Studies should employ rigorous statistical analyses alongside objective and consistent outcome measures to enhance reliability and comparability across literature.

## 5 Conclusion

This systematic review of 17 studies examining OMT as a treatment modality for feeding and swallowing dysfunction in children consistently demonstrated positive functional improvements with feeding and swallowing skills, though research design and quality were limited. There is a need for more consistent reporting of protocol design, increased sample sizes and a coordinated effort for better prospective study designs. Despite these limitations, the convergence of positive outcomes across diverse study designs reinforces the therapeutic potential of OMT and provides a compelling foundation for its continued clinical application.

## Author Contributions

**Danielle Carey:** conceptualization, methodology, supervision, writing – review and editing. **Robyn Merkel‐Walsh:** conceptualization, writing – review and editing, methodology, supervision. **Phoebe Burrage:** methodology, writing – original draft, data curation, formal analysis. **Melissa Hadlow:** writing – original draft, methodology, formal analysis, data curation. **Ghidaa Hasan A. Alrehaily:** writing – original draft, methodology, formal analysis, data curation. **Raymond J. Tseng:** conceptualization, writing – review and editing, methodology, supervision. **Sharon Smart:** conceptualization, writing – review and editing, methodology, formal analysis, data curation, supervision, project administration.

## Funding

The authors have nothing to report.

## Conflicts of Interest

Danielle Carey: Financial: Employee‐ North Carolina Tongue Tie Center, North Carolina. Clinical Instructor for: Medbridge, Summit Professional Education, Education Resources Inc. and Relias Therapy Education. Non‐financial: Board of Directors—International Consortium of oral Ankylofrenula Professionals (ICAP). ASHA CEA‐International Association of Orofacial Myology (IAOM). Robyn Merkel‐Walsh: Financial: Owner of Diamond Myo and Vocology and Mouths in Motion Mentoring. Author, lecturer and product developer—TalkTools. Employee—Ridgefield Board of Education Guest Faculty—The Breathe Institute Adjunct Faculty—Brescia University. Non‐Financial: Board Chair‐ Oral Motor Institute. Board Liaison Advisory Board, International Consortium of Ankylofrenula Professionals Volunteer CE Coordinator, Marshalla Speech and Language. Raymond J. Tseng: Financial: Owner/Director—North Carolina Tongue Tie Center, North Carolina. Non‐financial: Co‐Chair—ICAP Committee on Interprofessional Research, Education and Ankyloglossia Science (CIREAS). Sharon Smart: Non‐financial: Board Chair—Australasian Society of Tethered Oral Tissue (ASTOT). Co‐Chair—ICAP Committee on Interprofessional Research, Education and Ankyloglossia Science (CIREAS). Vice President of Academics—International Functional Speech‐Language Pathology Institute.

## Data Availability

The data that support the findings of this study are available from the corresponding author upon reasonable request.

## Appendix A Search Term and Boolean Operators for Initial Database Search

**Table:**

Population	Therapy	Target
Child* Adolescent* Teen* Paed* Ped* “Young children” “Young adult” “School age*” Preschool* Kinderg*	“Orofacial myo*” “Oral myo*” Oromyfuncation* “Myofunctional device*” Myofascial Myofunction*al “Myofunctional therapy” “OMT” “MFT” “Oral motor” “Oral placement”	Feed* “Dysphagia” Eat* Drink* Swallow* “Oropharyngeal difficult*” “Oropharyngeal dysphagia” Tongue “Oral mov*” “Oral intake” Masticat* Chew* Strength* “Bolus transport” “Bolus cohesion” “Oral transit” “Oral preparatory”

*Note:* ‘*’ represents unlimited truncation endings and spelling differences within/between databases. ‘“”’ represents phrase searching of the words contained within the double quotations.
